# Validation of a simple extraction procedure for bisphenol A identification from human plasma

**DOI:** 10.1371/journal.pone.0221774

**Published:** 2019-10-03

**Authors:** Idha Arfianti Wiraagni, Mustafa Ali Mohd, Rusdi bin Abd Rashid, Didi Erwandi bin Mohamad Haron

**Affiliations:** 1 Department of Pathology, Faculty of Medicine, University of Malaya, Kuala Lumpur, Malaysia; 2 Department of Forensic Medicine and Medicolegal, Faculty of Medicine, Universitas Gadjah Mada, Yogyakarta, Indonesia; 3 Shimadzu‐UM Center for Xenobiotics Studies (SUCXeS), Faculty of Medicine, University of Malaya, Kuala Lumpur, Malaysia; 4 Department of Psychological Medicine, Faculty of Medicine, University of Malaya, Kuala Lumpur, Malaysia; Bahauddin Zakariya University, PAKISTAN

## Abstract

The general population is exposed to bisphenol A (BPA) orally, parenterally, transdermally, and environmentally as a result of the use of BPA in food packaging, plastics, and personal care products. The majority of the population nowadays (91–99%) has detectable levels of BPA inside their body. In this study, we successfully performed an inexpensive, rapid, and simple protein precipitation procedure for extraction of BPA from human plasma, followed by analysis by LC-MS/MS. This method was specifically developed for handling large numbers of samples with minimum cost and volume of sample. The developed method was accurate, precise, and reproducible for quantification of BPA from human plasma samples in the concentration range of 10–2000 ng/mL. The method was performed on samples from 150 healthy volunteers who were enrolled in the study. The mean of observed BPA level was 2.22 ± 9.91 ng/mL. Higher BPA levels were observed for females compare to that of males (***p-*value = 0.002**), the BPA levels were higher in participants 33 years of age and older compared to those less than 33 years of age (***p-*value = 0.000**), then the BPA levels higher in subjects with tap water as source of drinking (***p-*value = 0.005**). This method may be valuable for general risk assessment of BPA for a large and varied population because of its efficiency and economical aspects.

## Introduction

The modern-day environment is filled with thousands of synthetic chemicals and compounds used in everyday life. Some of these chemicals are useful and beneficial to the human body while there are also numerous other chemicals that are known to be toxic and can cause undesirable effects in humans. Bisphenol (BP) is an industrial chemical that has been used to make certain plastics and resins since the 1960s. It is used in industry for increasing the thickness and durability of materials. BP analogues that were found in most environmental studies include BPA, BPB, BPF, BPS, and BPAF [1]. The global annual output of BPA is approximately 6.8 million tons [2]. BPA is commonly found in polycarbonate plastic, food storage containers, reusable drink containers, children's toys, and canned foods. In the pediatric population (infants and children), milk and beverage bottles provide ongoing daily exposure of BPA [3].

BPA is similar to endogenous estrogen which has the ability to bind to estrogen receptors, stimulating estrogen production and altering gonadotrophin hormone secretion [4]. These mechanisms can stimulate the development of endometriosis. Additionally, some studies found an association between urinary BPA concentrations and semen quality. Male Chinese workers with high BPA exposure (median urinary BPA concentration = 38.7 μg/L), had reduced sperm concentration, total count, and activity [5]. Research on male partners of infertile couples showed a positive correlation between BPA level in the urine and abnormal sperm morphology [6]. A study of 84 women undergoing *in vitro* fertilization (IVF) reported correlation between BPA level in urine (median = 2.6 μg/L) and reduced oocyte yield and peak serum estradiol [7]. One cross-sectional study with 192 female teenagers showed that there was a suggestive association between later onset of breast development and higher urinary BPA level [8]. Another study evaluating 367 pregnant women in their third trimester found a urinary BPA concentration of 1.3 μg/L. There was modest elevation in neonatal birth weight [9]. Additionally, a cohort study of 249 pregnant women (with 2.0 μg/L urinary BPA concentration) showed positive association between elevated maternal urinary BPA concentration and children with externalizing behaviors (such as aggression and hyperactivity) using the Behavioural Assessment System for Children-2 (BASC-2) [10]. Moreover, a study of 102 women showed a higher level of BPA in women with recurrent miscarriages compared to that of the control group [11]. A case-control study in Cyprus and Romania (n = 212) showed the effect of BPA as a thyroid disrupting chemical with influence on serum thyroid stimulating hormone [12]. In addition to the observed endocrine effects, animal models showed that BPA can induce hepatic cell mitochondria-mediated apoptosis, which may lead to chronic hepatotoxicity [13].

Because of its dangerous effects, human biologic monitoring (HBM) is very important for monitoring BPA levels in humans. Some studies have determined limit of detection (LOD) and limit of quantitation (LOQ), for detection of BPA from various matrices such as placental tissue, urine, serum, semen, blood, amniotic fluid, breastmilk, follicular fluid, and umbilical cord blood (Table 1). BPA can pass through the maternal-fetal placental barrier, thus, maternal and fetal serum, amniotic fluid, cord blood, and placental detection are suggested to evaluated the risk of fetal exposure to BPA [14]. Analytical techniques applied for measuring BPA in human matrices are GC-MS, LC-MS, LC-MS/MS, and enzyme-linked immunosorbent assay. Glucuronidase treatment is important for releasing glucuronic acid conjugation, which can then be followed by extraction by solid phase extraction (SPE), liquid-liquid extraction (LLE), stir bar sorptive extraction, or solid phase microextraction (SPME) [15].

**Table 1:** Analysis of bisphenol A (BPA) in human biological sample.

No	Sensitivity	Sample	Preparation	Detection	References
1	LOQ (0.1 ng/mL)	Urine	SPE	LC-MS/MS	[16]
2	LO (5 μg/L); LOQ (15 μg/L)	Urine, Plasma	Protein Precipitation	LC-MS/MS	[17]
3	LOQ (0.1 ng/mL)	Urine, Blood	SPE	HPLC–NESI–MS/MS	[18]
4	LOQ (0.01 ng/mL)	Urine, Serum	SPE	LC-MS/MS	[19]
5	LOD (1.7 ng/mL)	Urine, Cord Blood	LLE	UPLC/MS	[20]
6	LOD (0.01 ng/mL)	Blood	LLE	GC-MS	[21]
7	LOD (0.1 ng/mL)	Urine	SPE	GC-MS	[22]
8	LOQ (43.5 pg/mL)	Plasma, Seminal Plasma	LLE	LC-MS/MS	[23]

Some of the above studies have reported various extraction and detection methods of BPA. However, these methods require multiple steps and have onerous sample preparation. They predominantly used LLE and SPE to increase sensitivity, which require extra effort and time and are also quite costly. Both extraction techniques also require experienced personnel to perform them added costs if reproducing these methods as a part of routine examination [24]. Only one study used simple protein precipitation for extraction, but they had a less sensitive LOQ at 15 μg/L. BPA has become a global issue, and as such the monitoring report from all over the world, including developing countries, is extremely important for global assessment. Based on this risk assessment, the government can determine the best solution for the society. New extraction methods are needed to help solve these problems. Therefore, the aim of this study was to develop an inexpensive, rapid, and simple procedure for BPA extraction and detection from human plasma. This method gave higher sensitivity and accuracy by applying a simple protein precipitation extraction method. This extraction technique was found to be efficient and more economical than LLE and SPE.

## Materials and methods

### Materials and standards

The BPA ≥ 99% standard was purchased from Sigma-Aldrich (St. Louis, MO, USA). BPA-d16 was obtained from Cambridge Isotope Laboratories (Frontage Road Andover, MA, USA). All solvents and reagents used were of HPLC grade and purchased from Merck (Darmstadt, Germany). Control plasma samples were obtained from the UMMC blood bank Malaysia.

### Preparation of mobile phase, standards, quality control (QC), and calibration

#### Preparation of mobile phase

To prepare mobile phase A (2 mM of ammonium acetate in water), 154.16 mg of ammonium acetate was dissolved in 1 L of deionized water, filtered, degassed under vacuum, and the pH was measured with a pH meter (pH 6.7). Mobile phase B (methanol) was 1 L HPLC grade methanol filtered through a 0.2 μm membrane. This was followed by sonication under vacuum for the purpose of degassing.

#### Preparation of stock solution standards

To prepare the BPA stock solution, 2.0 mg of BPA was accurately weighed then dissolved into 2 mL methanol to obtain 1000 μg/mL.

#### Preparation of stock plasma standards

These were prepared by spiking 9900 μL aliquots of drug-free plasma with 100 μL of 1000 μg/mL BPA to give a concentration of 10 μg/mL. Aliquots for the QC samples were stored frozen at -20°C until further analysis.

#### Preparation of calibration curve and QC

All the calibrators and QC samples were prepared by spiking 10 μg/mL working standard solution into different volumes of plasma to obtain seven calibrators at 10, 20, 50, 100, 500, 1000, and 2000 ng/mL, and three QC samples at 70, 800, and 1500 ng/mL.

### Samples

A total of 150 blood samples were obtained from healthy volunteers that consenting to participation in the study. Demographic details such as gender, age, residential area, and ethnicity data were recorded. The inclusion criteria were 18 years of age or older, fully conscious, physically healthy, and no serious current psychiatric symptoms (i.e. psychotic episode). The exclusion criteria were refusal to participate, requiring advanced medical attention for a serious illness, and requiring psychiatric care for psychiatric symptoms. Institutional approval for the analysis of human samples was obtained from the Ethical Committee of University Malaya Medical Centre. Roughly 5 mL of human whole blood sample was collected into EDTA tubes. Cells were removed from plasma by centrifugation for 10 minutes at 2000 x g. Following centrifugation the liquid plasma was transferred into a clean polypropylene tube using a Pasteur pipette. The samples were stored at -20°C until they were analyzed.

### Extraction of plasma

Sample preparation was carried out using a protein precipitation extraction method. The frozen plasma was thawed at room temperature (25 ± 1°C). The thawed plasma was vortexed to ensure the sample was homogenous. To each 100 μL plasma sample, 50 μL of internal standard (IS) (containing 2 μg/mL of IS) was added, followed by the addition of 250 μL of acetonitrile (ACN). The mixture was vortexed, shaken for 5 s, and centrifuged for 2 min at 14800 rpm. The supernatant was filtered with a 0.2 μm syringe filter, then transferred to new a vial. Two microliters were then injected into the LC-MS/MS system (dx.doi.org/10.17504/protocols.io.3xrgpm6).

### Liquid chromatography apparatus and conditions

In this study, the LC system used consisted of an LC-20AD XR UFLC system with a SIL-HT automatic sample injector (Shimadzu, Kyoto Japan). The analytical column used was a Phenomenex, Gemini-NX C18 (150 mm length x 2.0 mm ID, particle size 5 μm) and Phenomenex, Gemini-NX C18 guard column (4 mm ID x 2.0 mm length). Column temperature was 40°C with a total running time of 11 min. Mobile phase used were 2 mM ammonium acetate (pH 6.7) in pump A and methanol in pump B. The flow rate was set at 0.35 mL/min and a gradient elution was used at room temperature. The gradient program began with 20% B, then ramped to 98% B at 6.00 min and held until 9.00 min. The gradient then returned to 20% B at 9.01 min and this condition was held until a total of 11.00 min. Sample injection volume was 2 μL.

### Mass spectrometry parameters

A Linear Ion Trap Quadrupole LC-MS/MS Spectrometer, QTRAP 5500, fitted with an ESI probe, and operated in the negative ionization mode was used to perform mass spectral analysis. The LC-MS/MS system was controlled by the Analyst software, version 1.6.3 (Applied Biosystems). Nitrogen was used as the nebulizer, auxiliary, collision, and curtain gas. Analytes were then quantified by multiple reactions monitoring (MRM). For quantitative analysis, MRM transitions of 227.119 → 211.700 and 227.119 → 133.000 for BPA were monitored with a dwell time of 20 ms. The mass spectrometry parameters for BPA were as follows: declustering potential (DP) of 150 V, collision energy (CE) of 34 V, and collision cell exit potential (CXP) of 13 V. The optimal conditions were as follows: ion source temperature of 450°C, ion spray voltage of 4500 V, curtain gas of 20.0 psi, collision gas of medium, ion source gas 1 of 35.0 psi, and ion source gas 2 of 35.0 psi. For BPA-d16, MRM transitions were 240.877 → 142.100 and had the same mass spectrometry parameters as BPA, except for a declustering potential of 210 V. Retention time of BPA was 5.07 and BPA-d16 was 5.02. Analyst 1.6.3 software was used for system control and data quantification.

### Validation

**Fig 1:**
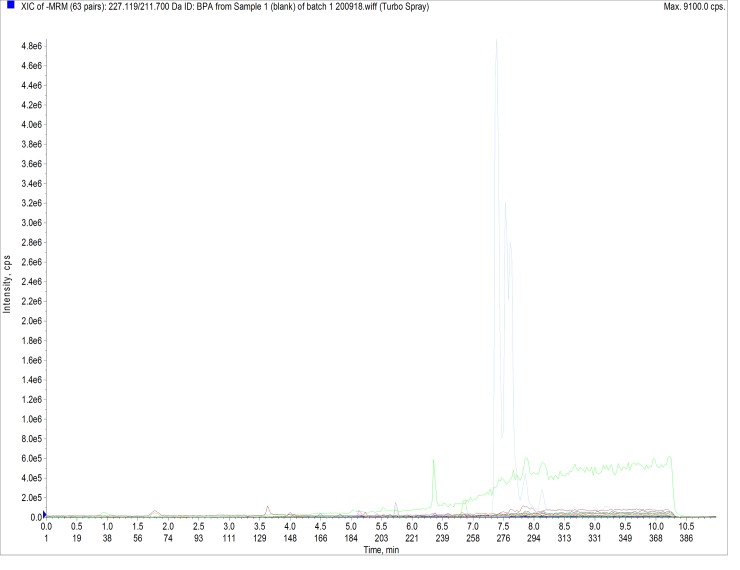
Representative MRM chromatogram of Blank.

**Fig 2:**
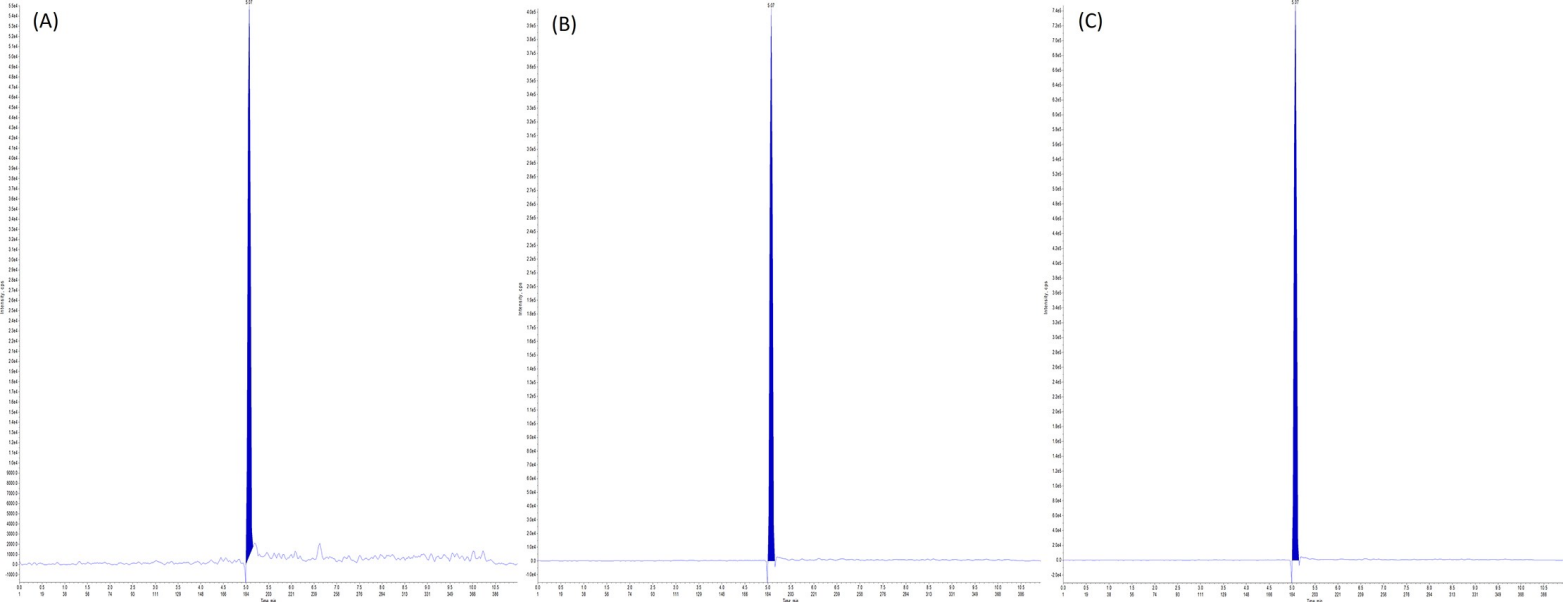
Representative MRM chromatogram of bisphenol A (BPA) in human plasma. RT: 5.07: (A) QCL intensity: 8.9e4; (B) QCM intensity: 7.0e5; (C) QCH intensity: 1.3e6.

In order to demonstrate the performance of the method and consistency of the analytical results, validation was performed to the United States Food and Drug Administration (USFDA) guidelines for bioanalytical method validation. Specificity was defined as non-interference between BPA and BPA-d16 using the proposed extraction procedure and LC-MS/MS conditions, and with no cross interferences at the retention time where BPA appears (Fig 1). Precision (include intraday and interday precision), accuracy, and recovery were determined by using 5 replicates of sample per concentration. Three concentrations of QC samples (spiked plasma) were used to build calculation, at the concentrations of 70, 800, and 1500 ng/mL (Fig 2). For stability, autosampler, freeze-thaw, and long-term stability were examined in this study.

## Results

### Linearity, sensitivity, and specificity

**Fig 3:**
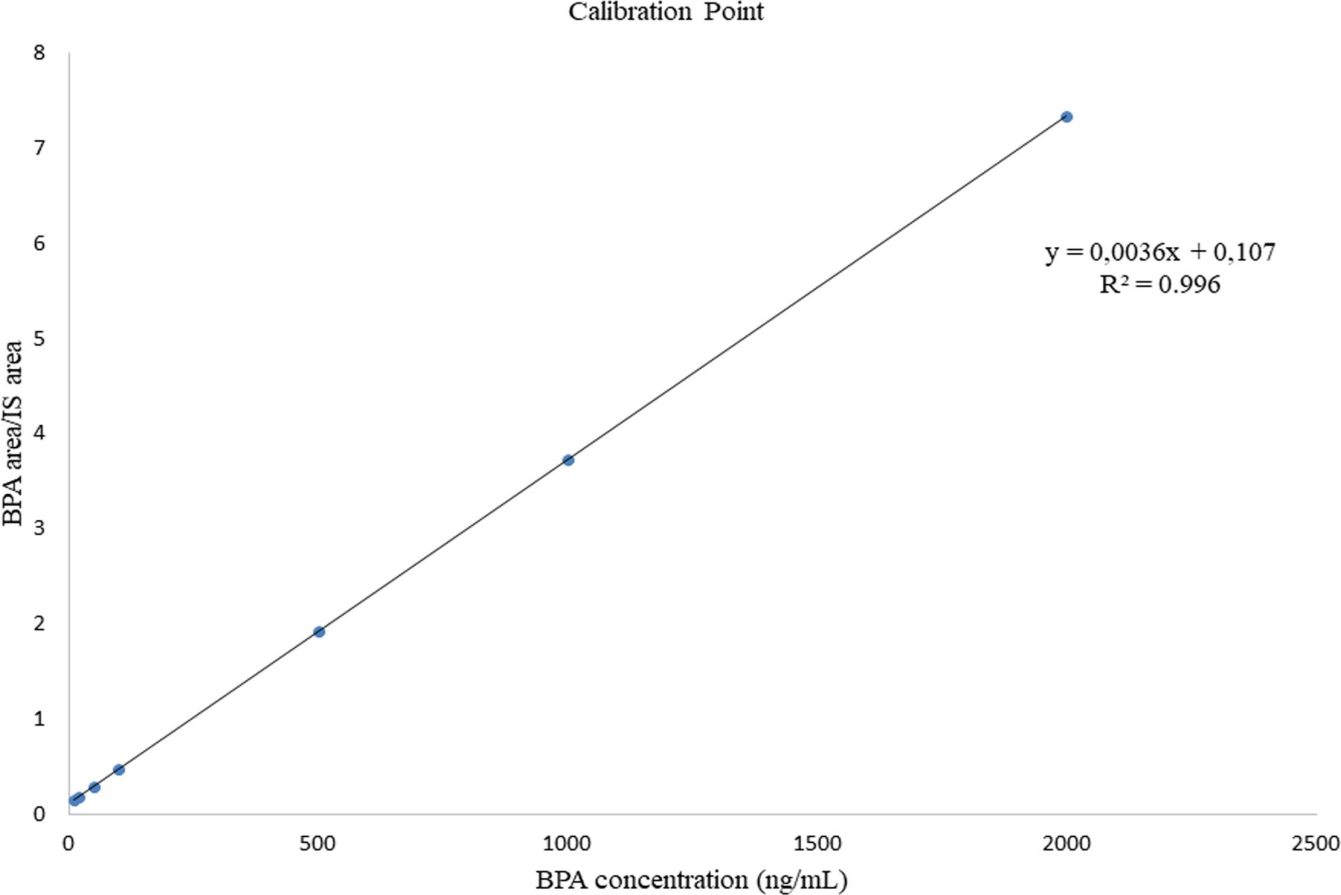
Calibration curves of BPA at different concentrations in human plasma.

No significant interfering peaks were observed from the retention time corresponding to BPA and the internal standards (Figs 1 and 2). Seven calibration points of 10, 20, 50, 100, 500, 1000, and 2000 ng/mL were used to evaluate the linearity of the standard calibration curve (Fig 3). The standard curve was linear with 1/X as weighing factor and reached good linearity (r > 0.997). The lower LOD was established at 5 ng/mL, while the lower LOQ was established at 10 ng/mL with an average signal to noise ratio which was greater than 10.

### Precision and accuracy

The intra- and interday precision and accuracy (%) data for BPA in plasma is summarized in Tables 2 and 3. Based on the mean percentage of the coefficient of variation (%CV) for three QC samples, the intra-day precision and inter-day precision of plasma sample ranged from 2.89% to 9.45% and from 6.49% to 11.01%, respectively. When assessed by means of the three QC samples, the accuracy for intra-day and inter-day for plasma sample ranged from 87.72% to 106.6% and from 93.7% to 98.58%, respectively. The results from this data clearly showed that the method that was developed has good accuracy, precision, and reproducibility for the quantification of BPA from human plasma.

**Table 2:** Intra-day reproducibility for BPA in human plasma.

Day	Quality Control	Average	SD	% CV	Accuracy (%)	Inaccuracy (%)
1	QCL (70 ng/mL)	74.68	2.45	3.28	106.6	6.6
	QCM (800 ng/mL)	755.8	21.84	2.89	94.48	5.52
	QCH (1500 ng/mL)	1426	105.26	7.38	95	5
2	QCL (70 ng/mL)	61.4	5.05	8.22	87.72	12.28
	QCM (800 ng/mL)	723	32.53	4.5	90.38	9.62
	QCH (1500 ng/mL)	1434	96.7	6.7	95.68	4.32
3	QCL (70 ng/mL)	62.82	4.69	7.47	89.76	10.24
	QCM (800 ng/mL)	770.2	72.78	9.45	96.32	3.68
	QCH (1500 ng/mL)	1576	134.65	8.54	105.08	5.08

QCL: quality control low; QCM: quality control medium; QCH: quality control high; SD: standard deviation; CV: coefficient of variation

**Table 3:** Inter-day reproducibility for BPA in human plasma.

Quality Control	Average	SD	% CV	Accuracy (%)	Inaccuracy (%)
QCL (70 ng/mL)	66.30	7.30	11.01	94.7	5.28
QCM (800 ng/mL)	749.67	48.68	6.49	93.7	6.2
QCH (1500 ng/mL)	1478.67	126.77	8.57	98.58	1.4

QCL: quality control low; QCM: quality control medium; QCH: quality control high; SD: standard deviation; CV: coefficient of variation

### Recovery

The recovery of BPA was tested at 10, 50, and 100 ng/mL and the mean recovery from human plasma was 84.6%, 91.67%, and 99.44%, respectively. These results indicated that the extraction efficiency of BPA using protein precipitation was quite good.

### Stability testing

The stability data for BPA is summarized in Tables 4, 5 and 6. The precision and accuracy for freeze-thaw stability in plasma ranged from 2.89% to 7.38% and from 94.48% to 111.32%, respectively (Table 4). The results indicated that the analyte was stable in plasma for two freeze-thaw cycles when stored at -20°C and thawed to room temperature. For autosampler stability the mean precision and accuracy ranged from 3.9% to 5.06% and from 91.46% to 100.08%, respectively, for plasma (Table 5). The results indicated that upon extraction, the BPA sample could be analyzed over 24 h in an autosampler at 20 ± 1°C with satisfactory precision and accuracy. BPA was stable in plasma after three months of storage at -20°C (Table 6). The results of stability testing showed that BPA was stable in plasma during storage, extraction, and testing.

**Table 4:** Freeze-thaw stability of BPA in human plasma.

Stability	Quality Control	Average	SD	% CV	Accuracy (%)	Inaccuracy (%)
Cycle 1	QCL (70 ng/mL)	74.68	2.45	3.28	106.6	6.6
	QCM (800 ng/mL)	755.8	21.84	2.89	94.48	5.52
	QCH (1500 ng/mL)	1426	105.26	7.38	95	5
Cycle 2	QCL (70 ng/mL)	77.96	5.56	7.13	111.32	11.32
	QCM (800 ng/mL)	804	35.55	4.42	100.46	0.46
	QCH (1500 ng/mL)	1582	105.69	6.68	105.42	5.42

QCL: quality control low; QCM: quality control medium; QCH: quality control high; SD: standard deviation; CV: coefficient of variation

**Table 5:** Autosampler stability of BPA in human plasma.

Stability	Quality Control	Average	SD	% CV	Accuracy (%)	Inaccuracy (%)
After 24 h	QCL (70 ng/mL)	64.02	2.5	3.9	91.46	8.54
	QCM (800 ng/mL)	745	37.30	5.01	93.14	6.86
	QCH (1500 ng/mL)	1500	75.83	5.06	100.08	0.08

QCL: quality control low; QCM: quality control medium; QCH: quality control high; SD: standard deviation; CV: coefficient of variation

**Table 6:** Long term stability of BPA in human plasma (stored three months at -20°C).

Stability	Quality Control	Average	SD	%CV	Accuracy (%)	Inaccuracy (%)
Long-term stabiliy	QCL (70 ng/mL)	73.74	4.77	6.47	105.36	5.36
	QCM (800 ng/mL)	707.8	9.78	1.38	88.48	11.52
	QCH (1500 ng/mL)	1336	36.47	2.73	89.14	10.86

QCL: quality control low; QCM: quality control medium; QCH: quality control high; SD: standard deviation; CV: coefficient of variation

## Discussion

### Extraction and separation conditions

Human plasma is a complex matrix rich in endogenous proteins and other biological compounds. ACN has previously been shown to be an extraction solvent that can produce significantly high recovery [25]. The use of ACN in the extraction method of this study gave good recovery and ruled out ion suppression. Good peak shape and high separation efficiency was obtained by the Phenomenex, Gemini-NX C18 (150 mm length x 2.0 mm ID, particle size 5 μm) and Phenomenex, Gemini-NX C18 guard column (4 mm ID x 2.0 mm length). Methanol was chosen as a mobile phase to prevent deterioration and broadening of peak shape [25]. This method also used ammonium acetate as an additive in order to produce better ionization, higher intensity, and better separation.

### Analysis of plasma samples

This method was applied to the screening of BPA levels in human blood plasma obtained from 150 healthy volunteers of Kuala Lumpur and Selangor, Malaysia (Table 7). Participants were an average of 30.03 ± 8.65 years of age, with the youngest being 20 and the oldest being 67 years old. The mean of height and weight were 158.26 ± 8.48 cm and 63.78 ± 17.89 kg, respectively.

**Table 7:** Baseline characteristics of participants.

Variable	N (%)	Variable	N (%)
Gender		Job Scope	
Male	43 (28.7)	University employee	106 (70.7)
Female	107 (71.3)	Hospital employee	42 (28)
Age Group		Bank employee	1(0.7)
<33 y	109 (72.7)	Student university	1(0.7)
≥33 y	41 (27.3)	BMI	
Race		Under weight	11 (7.3)
Melayu	134 (89.3)	Normal weight	76 (50.7)
Chinese	12 (8)	Pre obesity	35 (23.3)
Indian	4 (2.7)	Obesity	28 (18.6)

The observed levels of BPA ranged from 0 to 76.80 ng/mL with a mean concentration of 2.22 ± 9.91 ng/mL. We recorded the daily habits of each participants, such as smoking habit and plastic use, as well as their eating habits that may have correlation with BPA exposure. We performed statistical analysis to evaluated the significant differences of BPA level with respect to different daily habits and eating habits. The Independent Sample T test was conducted to compare the differences between two variables (Table 8). There were significant mean differences between BPA level correlated with gender, age, and source of drinking parameter (*p-*value = 0.002, 0.000, and 0.005 respectively). Higher BPA levels were observed for females compare to that of males, the BPA levels were higher in participants 33 years of age and older compared to those less than 33 years of age, then the BPA levels higher in subjects with tap water as source of drinking (Table 8).

**Table 8:** BPA level differences with respect to characteristics and daily habits.

Variable	N	Mean	*p-*value	Variable	N	Mean	*p-*value
Gender				Plastic use			
Male	43	0.29	**0.002**	Yes	131	2.28	0.599
Female	107	2.99		No	19	1.72	
Age				Source of food			
<33 y	109	0.847	**0.000**	Home cooking	91	2.79	0.072
≥33 y	41	5.852		Dining out	59	1.32	
Smoking				Source of drinking			
Yes	5	0.000	0.303	Tap water	111	2.882	**0.005**
No	145	2.292		Mineral Water	39	0.318	

The main mode of human exposure of BPA is oral, environmental, parenteral, and dermal exposure [26]. Canned food, coffee pots, soda cans, and other kitchen plastics are believed to be major sources of BPA via the oral route [27]. Over 500 peer-reviewed articles have reported BPA global distribution in environments such as: surface waters, tap waters, sediments, biosolids, air, soils, and wildlife [28]. Some parenteral sources are medical devices such as implants, catheters, tubing, hemolysis dialyzers, and some dental materials use BPA [29]. Additionally, BPA was found to be transferred via dermal exposure from personal care items and products containing bisphenols (thermal receipt paper) [30].

In this study, females were found to have higher BPA levels than males, possibly because of greater exposure to BPA. Small amounts of BPA are added to many personal care product such as cosmetics and hand sanitizer, that are regularly used by females [31]. Hand sanitizer and some products which had penetration enhancing chemicals, can increase the dermal absorption of lipophilic compounds such as BPA. One study reported high BPA levels in serum and urine after using hand sanitizer and then holding thermal receipt paper (high free BPA level intentionally applied to the outer layer) [30]. Moreover, there was the notion that more women enjoy shopping in stores and more worked as cashiers compared to men, which can increase the possibility of exposure via thermal receipt paper [32]. Additionally, females nowadays tend to consume fast food and canned food, resulting in poor diet quality, high prevalence of obesity, and high BPA exposure [33].

From a toxicokinetic study, BPA from the gastro-intestinal tract and parenteral routes were conjugated in the liver to non-toxic metabolites. The elimination of free BPA from the circulation via the renal system appears to be relatively fast. Because of this rapid conjugation and elimination, the measured BPA reflects recent exposure within the last several hours just prior to sample collection [29]. One study reported higher elimination rates among men, younger subjects, and those with increasing waist circumference (*p-*value = 0.013) [34]. These results were consistent with the finding of this study, because females and older participants (33 years old and above) had lower excretion rates, and thus, higher plasma BPA levels. Moreover, aging could lead to liver and renal dysfunction, thus enhancing vulnerability to acute organ injury [35]. The liver and renal system are target organs of BPA, as observed in several studies. As the function of either or both of these organs decline, BPA level in plasma increase [29].

## Conclusions

This study successfully employed an inexpensive, rapid, and simple procedure for BPA extraction and subsequent detection from human plasma by LC-MS/MS. The plasma extraction consisted of a simple protein precipitation method that consumes a low volume of organic solvent and trace volume of sample (100 μl). The method was suitable for identifying and effectively separating BPA with a lower quantification limit compared with previous studies, and had good precision and accuracy. This pilot study enrolled 150 healthy volunteers to evaluate BPA testing from plasma. Determination of BPA human plasma concentrations from the subjects could be satisfactorily performed by this proposed method. Our findings found a higher BPA level in females, older people, and using tap water as a source of drinking. Differences in exposure, metabolism, and organ function supported this result. Further research is needed on larger and varied cohort to explore the presence of BPA in the general population. This approach is particularly suited for building a general risk assessment database, especially for developing countries, owing to its high efficiency and low costs.

## Supporting information

S1 File Questionnaire BPA. (DOCX)
