# Supplementary material for: Validation of a simple extraction procedure for bisphenol A identification from human plasma
Source: PLoS One. 2019 Oct 3;14(10):e0221774. doi: 10.1371/journal.pone.0221774 (PMC6776257; doi:10.1371/journal.pone.0221774)
Supplement: S1 File — (DOCX) [file pone.0221774.s001.docx]

**Questionaire**

**Title of Study (*Tajuk Penyelidikan*)**: Monitoring of Substance Abuse and Toxic Chemicals in Selected Patient at UMMC *(Pemantauan Penyalahgunaan Ubat dan Toksik Kimia pada Pesakit Tertentu di UMMC)*

1. **Personal Information (*Maklumat Peribadi*)**

| Name (*Nama*) : |  | | |
| --- | --- | --- | --- |
| Gender (*Jantina*) : |  | Age (*Umur*) : |  |
| Weight (*Berat Badan*) (Kg) : |  | Height (*Ketinggian*) ( M) : |  |
| Race (*Bangsa*) : |  | | |
| Home Address (*Alamat Rumah*) : |  | | |

1. **Career / Education Information (*Maklumat Pekerjaan/Pendidikan*)**

| Occupation :  *(Pekerjaan)* : |  |
| --- | --- |
| Job scope (eg: education, service, manufacturing, etc) :  *(Tugas kerja*, *contoh : pendidikan, perkhidmatan, pengilangan, dll*) : |  |
| How long have you been working/studying in that place (year)?  *(Berapa lamakah anda telah bekerja/belajar di tempat tersebut?*) |  |

1. **Dietary Habits (*Tabiat Pemakanan*)**

**Please answer this column with tick √ as appropriate and fill the blank ! (*Tandakan √ dan tulis jawapan yang berkenaan*!)**

| 1. Which is your most favorite types of food? Choose one answer!   *(Apakah jenis masakan kegemaran anda?)* *Pilih satu jawaban!* | | Boiled (*Rebus*) | Fried (*Goreng*) | Raw (*Mentah*) |
| --- | --- | --- | --- | --- |
| 1. Where do you usually get your food? Choose one answer!   *(Di manakah biasanya anda mendapatkan makanan*?)  *Pilih satu jawaban!* | | Home Cooking  (*Masakan di rumah*) | Dining Out  (*Makan di luar*) | Take Away (*Beli dan bungkus*) |
| 1. What is your main source for drinking water? Choose one answer!*(Apakah sumber utama bagi air minuman anda?) Pilih satu jawaban!* | | Treated water  *(Air terawat)* | Bottled water  *(Air botol)* | River water  *(Air sungai)* |
| 1. How often do you eat this food in a week?   *(Berapa kerap anda makan makanan yang disenaraikan di bawah dalam seminggu*?) | | ≥ 4 times *(Kali)* | 1-3 times *(Kali)* | Never  *(Tidak pernah)* |
|  | Chicken (*Daging* *Ayam*) |  |  |  |
|  | Beef (*Daging* *Lembu*) |  |  |  |
|  | Lamb (*Daging* *Kambing*) |  |  |  |
|  | Pork (*Daging* *Khinzir*) |  |  |  |
|  | Fish (*Ikan*) |  |  |  |
|  | Seafood (*Makanan Laut*) |  |  |  |
|  | Chicken Eggs (*Telur Ayam*) |  |  |  |
|  | Others (*Lain-lain*), Please mention ! (*Senaraikan* !) | ..................................................... | | |

1. **Lifestyle Information (*Maklumat Gaya Hidup*)**

**Please answer this column with tick √ as appropriate and fill the blank ! (*Tandakan √ dan tulis jawapan yang berkenaan*!)**

| 1. Do you have/used any of this item in your house?   (*Adakah anda memiliki atau menggunakan peralatan/perkakas ini di rumah?*) | | Yes | No |
| --- | --- | --- | --- |
|  | Car (*Kereta*) |  |  |
|  | Non-stick cookware (*Perkakas memasak yang tidak melekat*) |  |  |
|  | Camping tents (*Khemah untuk perkemahan*) |  |  |
|  | Outdoor clothing (*Baju kalis air*) |  |  |
|  | Water/stain resistant carpet/fabric (*Karpet/fabrik kalis air & kotoran*) |  |  |
|  | Leather Sofa (*Sofa kulit*) |  |  |
|  | Dental floss/Denture cleaners (*Pembersih gigi*) |  |  |
|  | Cosmetic, eg: Nail polish, eye shadow, and blusher (*Kosmetik, contoh : Cat kuku, pewarna mata, dan pewarna pipi*) |  |  |
|  | Cutlery (*Perkakas makanan*)   1. Plastic (*Plastik*) 2. Glass (*Kaca*) 3. Ceramic (*Seramik*) |  |  |
| 1. How much time did you spend per day in your house (hours)?   (*Dalam sehari, berapa lamakah anda menghabiskan masa di rumah*?) | | ................................ | |
| 1. How much time did you spend per day in your car (hours)?   (*Dalam sehari, berapa lamakah anda menghabiskan masa di kereta?*) | | ................................ | |
| 1. How much time did you spend per day using computer (hours)   (*Dalam sehari, berapa lamakah anda menghabiskan masa di depan komputer?*) | | ................................ | |
| 1. Do you smoke? (*Apakah anda merokok?*)   If yes, how many cigarettes you smoke per day?  (*Jika ya, berapakah batang rokok yang anda hisap dalam sehari?*) | |  |  |
|  |  | ................................ | |

Thank You for your corporation (*Terimakasih atas kerjasama*)
